# SPAM—sub partual analgesia with meptazinol: a prospective cohort study comparing intramuscular with intravenous administration

**DOI:** 10.1007/s00404-023-07056-y

**Published:** 2023-05-09

**Authors:** Katharina Germeshausen, Aissa Linzbach, Janine Zöllkau, Yvonne Heimann, Ekkehard Schleussner, Tanja Groten, Friederike Weschenfelder

**Affiliations:** Department of Obstetrics, University Hospital Jena, Am Klinikum 1, 07747 Jena, Germany

**Keywords:** Meptazinol, Obstetrics, Pain therapy, Labor pain, Side effects, Application, Questionnaire, Pain measurement, Vaginal birth

## Abstract

**Purpose:**

Safe and effective analgesia sub partu is one of the central issues in optimizing vaginal delivery birth experiences. Meptazinol is a common opiate approved for treating labor pain in the first stage of labor. According to the manufacturer, manual meptazinol can be applied intramuscularly or intravenously. The aim of this study was to compare the two application methods in terms of efficacy in pain relief, occurrence of side effects and treatment satisfaction.

**Methods:**

132 patients with singleton term pregnancies and intended vaginal delivery, receiving meptazinol during first stage of labor were included in this prospective cohort study from 05/2020 to 01/2021. We evaluated effectiveness in pain relief and treatment satisfaction using numeric rating scales (NRS) and documented the occurrence of adverse effects. Chi-square test or Fisher exact test were used to compare categorical data and Mann–Whitney *U* test to compare continuous data between the two treatment groups. Statistical analysis was done by SPSS 27.0. A *p* value < 0.05 was considered to indicate statistical significance (two tailed).

**Results:**

Meptazinol decreased labor pain significantly from a NRS of 8 (IQR 8–10) to 6 (IQR 4.75–8) in both treatment groups with no difference in effectiveness between the groups. Frequency of effective pain reduction of a decrease of 2 or more on the NRS did not differ between groups (39.4% vs 54.5%, *p* = 0.116), as the occurrence of adverse effects. 12% of the newborns were admitted to NICU, the median NApH was 7.195.

**Conclusion:**

Meptazinol significantly reduces labor pain regardless of the method of application: intramuscular or intravenous. According to our data, no preferable route could be identified. The comparably poorer perinatal outcome in our study cohort hinders us to confirm that meptazinol is safe and can be recommended without restrictions.

**Supplementary Information:**

The online version contains supplementary material available at 10.1007/s00404-023-07056-y.

## What does this study add to the clinical work

**Table:** 

Our study demonstrates an equieffective potential of meptazinol regardless of the application route chosen and provides methodical data on how to measure pain relief and patient satisfaction in the context of spontaneous labor. Furthermore, our study suggests a reevaluation of the use of opioids during labor reporting comparable low perinatal outcome in a low risk cohort.	

## Introduction

Labor pain is the most intense pain women experience in their lives [1] and pain management during labor is an important and central component when caring for childbearing women. Adequate pain management during childbirth reduces stress and increases satisfaction with the birth experience [2, 3], an increasing important issue in high-income countries [4]. Up to 80% of women choose pharmacological options for pain relief during labor. Neuraxial techniques such as epidurals are most effective at providing labor analgesia and are considered to be the gold standard of pain relief during labor and delivery [5, 6]. In Germany, in 2017, the rate of epidurals during labor was 23% and in 20% non-neuroaxial procedures were applied [7], which is within the international range reported to be between 10 and 83%. Besides inhalative nitrous oxide (N_2_O), systemic opioids including meptazinol, pethidine, fentanyl, remifentanil and alfentanil are used either to avoid or delay using neuraxial analgesia or in cases of contraindication and application failure [8]. Additionally and in combination with opioids, drugs with spasmolytic effects (e.g. butylscopolamine) are commonly used to relief pain during early stages of labor. Systemic opioids are associated with numerous adverse side effects like nausea and vomiting, respiratory depression, decreased fetal heart rate variability as well as respiratory depression and neurobehavioral changes in the newborn. Repeated doses of systemic opioids administered during the course of labor may thus result in decreased Apgar scores, neonatal respiratory depression, and impaired early breastfeeding [8].

Meptazinol is a partial μ1-opioid receptor agonist with low affinity to the µ2-opioid receptor mediating analgesic effects by central cholinergic effects while the effect on respiratory depression is rather low [9]. It can be administered intravenously and intramuscularly in equieffective weight adapted dosage and effect [10]. In a clinical review summarizing studies on parenteral administration, the authors describe meptazinol as a centrally acting drug which produces analgesia of comparable intensity to opiates and report first evidence that the analgesic effect is more rapid in onset and shorter in duration [11]. The reported incidences of side effects are similar to other opiates, except that the incidence of central nervous effects such as euphoria, dysphoria and hallucinations were rare. The authors concluded that meptazinol is particularly suitable for the use in obstetrics [11]. Additionally, it was demonstrated that meptazinol has a rapid bioavailability, a short half-life of two hours, a wash out time of less than six hours and did not accumulate in the body irrespective of the application method [12, 13]. The favorable side effect profile was confirmed by concurrent studies reporting the most commonly described side effects to be of gastrointestinal nature accompanied by drowsiness and dizziness [14]. Thus, meptazinol could be regarded to be a potent and safe analgesic suitable to be offered during labor and delivery and it was widely introduced to German labor wards since the 1980s. Although no further research was performed on the safety and effectiveness of meptazinol during labor, according to a survey in 2007, still approximately 30% of obstetric units in Germany regularly offered and administered meptazinol [15, 16]. The first clinical study on the use of meptazinol during labor was performed in 2016. Singer and coauthors compared the effectiveness of pethidine and meptazinol on labor pain in a retrospective approach and demonstrated an advantage of meptazinol being associated with less need for secondary regional analgesia [17]. While in their study, meptazinol was usually administered intravenously (83%) [17], at our institution meptazinol was given solely intramuscularly. In this study, we aimed to investigate whether we could achieve equally effective pain relief and perinatal outcome by intramuscular and intravenous administration of meptazinol, without causing substantial increase in adverse effects.

## Methods

### Cohort composition

This study is a prospective observational single-center cohort study, which was conducted at a tertiary care center between May 2020 and January 2021. From May 2020 to September 2020, meptazinol was administered intramuscularly (subgroup: Meptazinol^IM^) followed by a second period of 5 months from September 2020 until January 2021 when meptazinol was administered intravenously (subgroup: Meptazinol^IV^). We consecutively included all women who received meptazinol during labor, to achieve a number of more than 50 cases in each group. Inclusion criteria were singleton term pregnancies (gestational age >  = completed 37 weeks), presentation in 1^st^ stage of labor, intended vaginal delivery, and fetal wellbeing confirmed by cardiotocogram (classified as normal following the FIGO-Score) at study inclusion. All participants were older than 18 years and gave informed consent. Exclusion criteria were application of meptazinol before study inclusion, permanent intake of pain medication, demand for non-neuraxial pain treatment at study inclusion and maternal or fetal conditions constituting contraindications against meptazinol.

### Application methods and dosages

All patients were routinely educated about pain management options upon admission to the delivery room. For both application methods, recommended weight-adapted dosage with 2 mg meptazinol per kg body weight according to the manufacturer’s manual was used [10]. The Meptazinol^IM^ subgroup received weight-adapted undiluted dosage of meptazinol intramuscularly, while the Meptazinol^IV^ subgroup received weight-adapted dosage diluted in 250 ml sodium chloride 0.9% over a 30 min period usually once during first stage of labor.

If demanded by the patient, a consecutive dosage of meptazinol was administered after a time period of 2–4 h, as recommended by the manufacturer’s information.

### Outcome parameters

Primary outcome measures were the effect on pain reduction and the incidence of side effects. We developed the SPAM case report form (SPAM-CRF) to document the effect on pain relief and the occurrence of side effects (see Supplement S1). Actual pain was assessed by the midwives using a numeric rating scale (NRS) from 0 to 10, with 0 being no pain and 10 representing worst imaginable pain and documented accordingly. Pain reduction (Δ) was calculated using NRS scores before and after application of meptazinol. Pain reduction of more than two points on the NRS scale (pain reduction Δ > 2) was considered as effective treatment according to previous publications [18, 19]. Occurrence of side effects within the first two hours after application was documented by the midwife in charge, according to the SPAM-CRF (Supplement S1: SPAM-CRF). Following delivery, before the patient was transferred from the delivery room to the ward, an additional (third) assessment of pain was done by the midwives.

Inclusion and exclusion parameters, the route of administration, dosage of meptazinol and possible confounders like of cervical ripeness (Bishop Score), CTG scores additional medication applied as well as outcome data (mode of delivery, time of birth, administration of oxytocin, neonatal weight, head circumference) were documented on the CRF. As secondary outcomes parameters to be analyzed, we defined perinatal outcome, additional pain medication and the application of dimenhydrinate to treat nausea and emesis.

### “QUIPS-Geburt” questionnaire

As part of the routine care in our institution, all participants received the “QUIPS-Geburt” questionnaire of the benchmarking project QUIPS (Quality-Improvement-in-Postoperative-Pain-Management) 24–48 h following delivery. This patient-reported outcome questionnaire assesses patient pain perception and pain treatment following operative interventions and has recently been validated to record pain encountered during vaginal delivery [20, 21] (Link: https://www.quips-projekt.de/uber-quips-geburt).

### Statistical analysis

Chi-square test or Fisher exact test was used to compare categorical data between the two groups: Meptazinol^IM^ group (*n* = 66) and Meptazinol^IV^ group (*n* = 66). Since most of the continuous data were not normally distributed, we used the median and interquartile range for data presentation and description. Mann–Whitney *U* test was performed to compare continuous data between the two treatment groups. The Wilcoxon signed-rank test was used to compare the pain scores before and after administration of meptazinol. A *p* value < 0.05 was considered to indicate statistical significance (two tailed). Since this was an observational clinical study, no prior sample size calculation was performed. The program utilized for the statistical analysis was SPSS 27.0.

We do confirm that any research activities during this study were performed according to ethical standards of the Declaration of Helsinki and the protocol for this research project has been approved by the Ethics Committee of the Friedrich-Schiller-University, Jena, Germany (Reg.-Nr. 2020-1745-Daten).

## Results

**Fig. 1:**
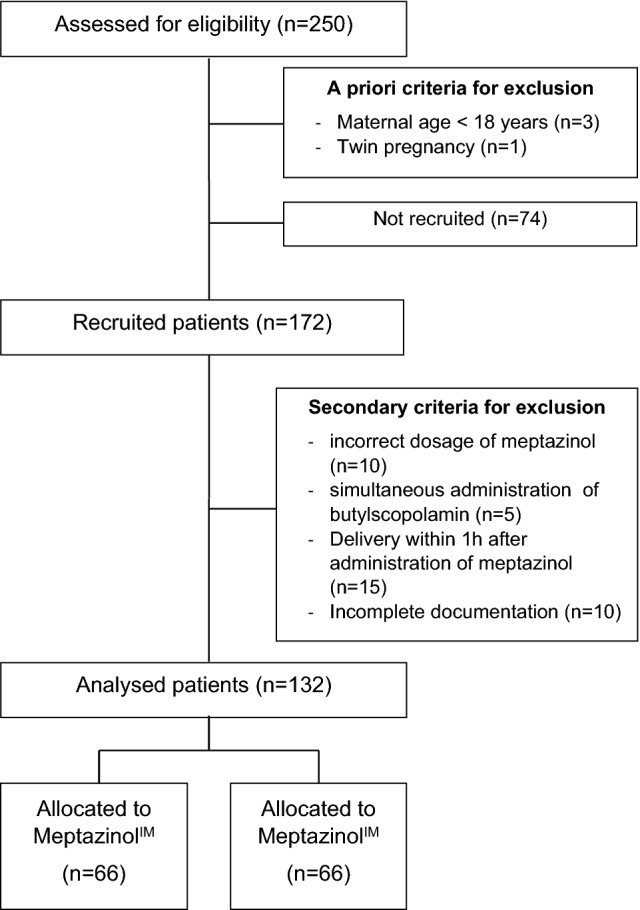
Cohort composition

During the study period, 250 patients received meptazinol, four had to be excluded (one twin pregnancies and three because of maternal age < 18 years). From the remaining 246 women, 172 women consented to participate in the study. 40 cases needed to be excluded from the analysis due to study protocol irregularities (10 incorrect dosage of meptazinol, 5 cases with simultaneous administration of butylscopolamine, 15 cases where delivery occurred within 1 h meptazinol administration and 10 cases due to incomplete data). The cohort composition is depicted in Fig. 1.

Study group characteristics and perinatal outcome data are presented in Table 1. There were no relevant statistically different demographic or outcome data between the study groups. There was no difference concerning mode of delivery, spontaneous vaginal delivery was 62.1% in the Meptazinol^IM^-group and 68.2% in the Meptazinol^IV^-group (*p* = 0.584) with corresponding C-section rates 19.7% vs. 5.2% (*p* = 0.647). We did not find differences concerning maternal and neonatal outcomes.

**Table 1:** Cohort characteristics and perinatal outcome: univariate analysis of the subgroups Meptazinol^IM^ (*n* = 66) and Meptazinol^IV^ (*n* = 66)

Demographic data	Meptazinol^IM^ (*n* = 66)	Meptazinol^IV^ (*n* = 66)	*p*
Age (years)	30 (27–32.3)	30 (24.8–34)	0.889
BMI (kg/m^2^)	29.8 (27.6–33.1)	29.4 (26.9–33.5)	0.631
Gestational age at delivery (days)	279 (272–283)	281.5 (276–284)	0.06
Bishop score	7 (5–10)	8 (7–10)	0.064
IOL	19 (28.8%)	21 (31.8%)	0.850
Primiparous	47 (71.2%)	54 (81.8%)	0.218
Multiparous	19 (28.8%)	12 (18.2%)	0.218
*Obstetric outcome*			
*Mode of delivery*			
Spontaneous vaginal	41 (62.1%)	45 (68.2%)	0.584
Vaginal operative	12 (18.2%)	11 (16.7%)	1
C-section	13 (19.7%)	10 (15.2%)	0.647
*Length of labor (minutes)*			
1st Stage	430 (285–665)	465 (286.3–708.3)	0.489
2nd Stage	87 (38.5–145.5)	57 (28.5–95.8)	0.094
Interval between Meptazinol and Birth (minutes)	254 (152.5–468)	215.5 (144.75–441)	0.438
Oxytocin for augmentation of labor	43 (65.2%)	39 (59.1%)	0.591
Episiotomy	12 (18.2%)	21 (31.8%)	0.107
*Neonatal outcome*			
Birthweight (g)	3485 (3120–3867)	3570 (3175–3896)	0.503
Head circumference (cm)	35 (33.5–35.5)	35 (34–36)	0.641
APGAR 1	9 (8–9)	9 (8–9)	0.438
APGAR 5	9 (9–19)	9 (9–19)	0.150
APGAR 10	10 (9–10)	10 (10–10)	0.052
pH umbilical artery	7.195 (7.15–7.26)	7.195 (7.14–7.26)	0.956
Respiratory distress syndrome	8 (12.1%)	7 (10.6%)	1
CPAP	8 (12.1%)	7 (10.6%)	1
NICU admission	6 (9.1%)	8 (12.1%)	0.778

Data are *n* (%) or median and interquartile range (IQR) unless otherwise specified. Significant findings (*p* < 0.05) are highlighted in bold. Bishop Score—pre-labor scoring system to evaluate cervical readiness based on dilatation, effacement, station and cervical consistency and position; *BMI* body mass index, *CPAP *continuous positive airway pressure, *IOL* induction of labor; length of labor—1st stage (dilating stage until 10 cm dilatation) and 2nd stage (10 cm dilatation to delivery of the fetus), *Meptazinol*^*IM*^ subgroup with intramuscular application of meptazinol, *Meptazinol*^*IV*^ subgroup with intravenous application of meptazinol, *NICU* neonatal intensive care unit

### Pain relief and side effects

Both groups had a median NRS pain score of eight before application of meptazinol, with a statistical difference (*p* = 0.035). There was a significant reduction of labor pain from a median of 8 (IQR 8–10) to 6 (IQR 4.75–8) within one hour following application in the Meptazinol^IM^ group and from a median of 8 (IQR 7–9) to a median of 5 (IQR 4–6.25) in the Meptazinol^IV^ group (Table 2).

**Table 2:** Primary outcome data on pain reduction and side effects: univariate analysis of the subgroups Meptazinol^IM^ (*n* = 66) and Meptazinol^IV^ (*n* = 66)

	Meptazinol^IM^ (*n* = 66)	Meptazinol^IV^ (*n* = 66)	*p*
Meptazinol dose in mg	160 (140–180)	160 (140–180)	0.631
*Pain score (NRS)*			
Before application	8 (8–10)	8 (7–9)	**0.035**
1 h after application	6 (4.75–8)	5 (4–6.25)	**0.026**
Δ pain reduction	2 (1–4)	3 (1.75–4)	0.181
Effective pain reduction (Δ > 2)	26 (39.4%)	36 (54.5%)	0.116
*Side effects*			
Nausea	23 (34.8%)	29 (43.9%)	0.373
Emesis	13 (19.7%)	23 (34.8%)	0.078
Fatigue	13 (19.7%)	13 (19.7%)	1
Vertigo	23 (34.8%)	23 (34.8%)	1
Perspiration	9 (13.6%)	12 (18.2%)	0.635
Headache	1 (1.5%)	3 (4.5%)	0.619
Intolerance	0 (0%)	1 (1.5%)	1
Satisfaction with pain treatment (NRS)	7 (5.25–9)	6 (5–8)	0.162

Data are *n* (%) or median and interquartile range (IQR) unless otherwise specified. Significant findings (*p* < 0.05) are highlighted in bold. Effective pain reduction, defined as pain reduction Δ > 2 after Meptazinol, Meptazinol^IM^ – subgroup with intramuscular application of meptazinol, Meptazinol^IV^ subgroup with intravenous application of meptazinol, *NRS* numeric rating scale

**Fig. 2:**
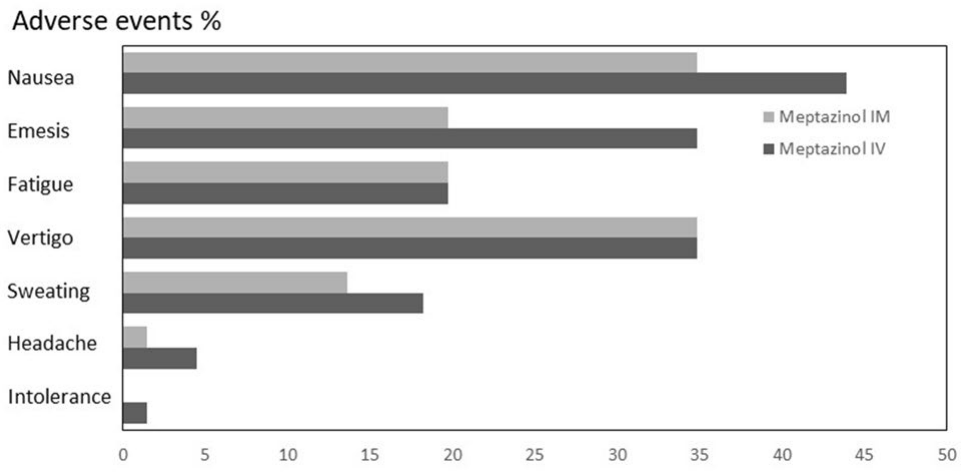
Comparison of side effects (%) between the two groups: intramuscular (Meptazinol^IM^) and intravenous (Meptazinol.^IV^)

Pain reduction (Δ NRS score before application and NRS score one hour following application) in the Meptazinol^IM^ group was 2 (IQR 1–4) points compared to 3 (IQR 1.75–4) points in the Meptazinol^IV^ group (*p* = 0.181). Occurrence of effective pain reduction (defined by pain reduction Δ > 2) did not differ significantly between both groups (39.4% in the Meptazinol^IM^ group and 54.5% in the Meptazinol^IV^ group) (*p* = 0.116). We could not find significant differences regarding side effects (Fig. 2).

None of the women in both groups received a repeated application of meptazinol. The need for further alternative pain medication did not differ between groups concerning non-opioids, opioids and regional anesthesia. LIVOPAN® usage was significantly higher in the Meptazinol^IM^ subgroup (27.3%) compared to Meptazinol^IV^ with 10.6%. In 19.7% of the cases in the Meptazinol^IV^ group, dimenhydrinate was administered for nausea and emesis control, which was significantly more frequent compared to the Meptazinol^IM^ group with 6.1% (*p* = 0.035) (Table 3).

**Table 3:** Data on additional medication: univariate analysis of the subgroups Meptazinol^IM^ (*n* = 66) and Meptazinol^IV^ (*n* = 66)

	Meptazinol^IM^ (*n* = 66)	Meptazinol^IV^ (*n* = 66)	*p*
*Additional pain medication*			
Non-opioids	18 (27.3%)	20 (30.3%)	0.848
Livopan	18 (27.3%)	7 (10.6%)	**0.025**
Fentanyl	4 (6.1%)	4 (6.1%)	1
Regional anaesthetics	27 (40.9%)	27 (40.9%)	1
*Combination of pain medication:*			
Meptazinol alone	20 (30.3%)	21 (31.8%)	1
Meptazinol + one other medications	26 (39.4%)	33 (50%)	0.294
Meptazinol + two other medications	19 (28.8%)	11 (16.7%)	0.145
Meptazinol + three other medications	1 (1.5%)	1 (1.5%)	1
*Combination of pain medication (without Livopan)*:			
Meptazinol + one other medication	27 (40.9%)	33 (50%)	0.382
Meptazinol + two other medications	11 (16.7%)	9 (13.6%)	0.809
Use of dimenhydrinate (%)	4 (6.1%)	13 (19.7%)	**0.035**

Data are *n* (%) unless otherwise specified. Significant findings (*p* < 0.05) are highlighted in bold. Meptazinol^IM^ – subgroup with intramuscular application of meptazinol; Meptazinol^IV^ subgroup with intravenous application of meptazinol

### Satisfaction with pain treatment

Satisfaction with pain treatment was evaluated using the NRS scale during the study about two hours following delivery and additionally using the QUIPS questionnaire validated for vaginal delivery within 24–48 h following delivery.

Asked by the midwives two hours following delivery when leaving the delivery ward, participants of both groups stated moderate to high satisfaction scores (7 (IQR 5.25–9 in the Meptazinol^IM^ group and 6 (IQR 5–8) in the Meptazinol^IV^ group (*p* = 0.162)) on the NRS. (Table 2).

Results of the QUIPS questionnaire were available from 78 patients 38 patients of the Meptazinol^IM^ group and 40 patients of the Meptazinol^IV^ group (*p* = 0.860). Reasons for missing Quips results were discharge before 24 h and refusal to fill out the questionnaire. Both groups reported high pain scores during labor with a median of 9.5 (IQR 9–10) in the Meptazinol^IM^ group and 10 (IQR 9–10) in the Meptazinol^IV^ group (*p* = 0.632). More than 90% of all women (Meptazinol^IM^: 92.1%, Meptazinol^IV^: 92.3%) reported a pain reduction by the medication applied reaching a median pain score of 7 (IQR 5–8) in the Meptazinol^IM^ group and 6 (IQR 5–8) in the Meptazinol^IV^ group (*p* = 0.898).

## Discussion

In our study cohort of 132 women, application of meptazinol showed a significant reduction of labor pain in both treatment groups demonstrating an equipotent effect in pain relief for the intravenous and intramuscular application. The rate of participants reaching an effective reduction in pain of more than two points on the NRS did not differ between groups. In addition, no significant group differences could be observed regarding the occurrence of adverse effects or satisfactions with the pain treatment. Thus, this small prospective observational study clearly demonstrates that the application of meptazinol intravenously or intramuscularly to be equieffective.

Pain scores were significantly lower in the Meptazinol^IV^ group before and following medication. However, the retrieved delta in pain reduction did not differ between the groups. Since observed NRS were lower in the Meptazinol^IV^ group before and following drug application, these differences might be rather explained by group characteristics than by the route of drug administration. Participants in the Meptazinol^IV^ group might have had a different pain tolerance, or asked for pain relief at an earlier stage of labor [22].

Previous studies considered a minimum of a two-point decrease on the NRS to represent a clinically relevant reduction in pain [18, 19, 23]. Accordingly, pain reduction (Δ) was calculated from the NRS values before and one hour after application of meptazinol and pain treatment was considered effective, if more than two points reduction on the NRS scale was reported (pain reduction Δ > 2). There was no difference between the Meptazinol^IM^ (2; IQR 1–4) and Meptazinol^IV^ (3; IQR 1.75–4) group in the calculated delta of NRS. However, the rate of participants retrieving an effective pain reduction was higher in the Meptazinol^IV^ group (54.4% vs. 39.4%) but the difference did not reveal to be statistical significant (*p* = 0.116). Since participants in the Meptazinol^IV^ group started from lower NRS values, corresponding to a lower level of pain at the time receiving meptazinol, this might be a reason for reaching a more profound effect in reduction of pain.

Observed adverse effects including nausea, emesis and vertigo were similar in both groups. (Fig. 2) Although not meeting statistical significance it is worth noticing that emesis occurred in 34.8% in the Meptazinol^IV^ group compared to 19.7% in the Meptazinol^IM^ group. Consistently, we observed a significantly higher rate of dimenhydrinate application in the Meptazinol^IV^ group to treat nausea and emesis. However, the higher rate of dimenhydrinate application could be in part explained by the care givers assuming nausea and vomiting to occur in higher frequencies following i.v. application, which might have led to a more active offering of antiemetic therapy to the participants by the midwifes. In the literature, a history of hyperemesis or motion sickness are described to be risk factors for medication-induced emesis [24]. Since we did not collect data on history of hyperemesis or motion sickness, we were not able to control our results for this confounder.

Although the percentage of patients reporting an effective release in pain was higher in the Meptazinol^IV^ group, we could not observe a corresponding difference in treatment satisfactions. Actually, the Meptazinol^IV^ group did show slightly lower scores in treatment satisfaction, which might correspond to the higher occurrence of emesis in this group. This demonstrates that treatment satisfaction might not only be dependent on the pain relief retrieved. Besides accompanying side effects of given medication further cofounding factors including the involvement of the patient in decision making, the relationship with the caregivers, the retrieved mode of delivery and the neonatal outcome influences the perception of treatment satisfaction [25, 26].

Since the SPAM data collection did not include a specific survey on these possible confounders, we decided to include data from QUIPS, a routinely performed evaluation of patient satisfaction. The QUIPS questionnaire, evaluating the satisfaction with pain treatment during vaginal birth also includes questions about the contentment with the midwife [20]. Results retrieved from the QUIPS questionnaire demonstrate a high treatment satisfaction reaching a median of 10 in both groups (Meptazinol^IM^ IQR 9.75–10; Meptazinol^IV^ IQR 9–10; *p* = 0.204) (Table 4). However, since we did not observe any differences in the QUIPS results concerning satisfaction with the treatment and contentment with the midwife (Supplement S2) between the two treatment groups, we do not consider these confounders to be of an impact on our study results. Accordingly, the clinical outcome parameters did not differ between groups and are thus not likely to impact on our study results.

**Table 4:** Selected study outcome data of the QUIPS questionnaire (*n* = 78): univariate analysis of the subgroups Meptazinol^IM^ (*n* = 38) and Meptazinol^IV^ (*n* = 40)

QUIPS study outcome data	Meptazinol^IM^ (*n* = 38)	Meptazinol^IV^ (*n* = 40)	*p* value
Pain score during birth*	9.5 (9–10)	10 (9–10)	0.632
Pain medication used	38 (100%)	39 (97.5%)	0.327
Relief after pain medication	35 (92.1%)	36 (92.3%)	0.974
Pain score after administration	7 (5–8)	6 (5–8)	0.898
Contentment with midwife	10 (9.75–10)	10 (9–10)	0.204
Satisfaction with pain treatment	8 (6.75–9)	8 (6–9)	0.879
More pain medication desired than administered	15 (40.5%)	14 (35.9%)	0.677
Δ pain reduction	2 (2–3)	3 (1–4)	0.543
Pain reduction after pain medication > 2 NRS*	28 (77.8%)	27 (69.2%)	0.403

Data are *n* (%) or median and interquartile range (IQR) unless otherwise specified. Significant findings (*p* < 0.05) are highlighted in bold. Cohort differs due to missing cases (*n* = 75). Meptazinol^IM^ subgroup with intramuscular application of meptazinol, Meptazinol^IV^ subgroup with intravenous application of meptazinol, *NRS* numeric rating scale

However, despite lacking group differences in our cohort of low-risk pregnancies at term with intended vaginal birth, we observed a C-section rate of 17.4%, of assisted vaginal delivery of 17.4%, a rate of episiotomies of 35% and 62% received oxytocin to augment labor. Additionally, the median UA pH was 7.19 and 11% of the children were admitted to NICU. For the year 2020, when the study was conducted, our hospital records revealed, in the low-risk cohort of more than 37 weeks of pregnancy with intended vaginal birth, a C-section rate of 14.61%, a rate of assisted vaginal delivery of 8.4%, a rate of episiotomies of 12.4%, a median UApH of 7.22. 6.4% of the newborns were admitted to NICU and 42.3% of the mothers received oxytocin to augment labor. Comparing that data, we can clearly not confirm the safety of meptazinol application for pain reduction during labor. Since this was not the aim of our study we did not perform statistical calculation on this data; however, a doubling NICU admission rate, the comparable low UApH combined with a doubling in episiotomies, a procedure which, at our institution, is only performed in cases where fetal distress demands a prompt delivery, strongly indicates an increase in fetal distress in low-risk women receiving meptazinol during labor.

### Limitations of this study

The aim of our study was to compare the effectiveness in pain relief and the occurrence of adverse side effects after the application of meptazinol either intravenously or intramuscularly. In the absence of preliminary data, we were not able to perform a power analysis in advance. Thus, in our small study, the case number was probably too low to detect significant differences in the occurrence of side effects. However, the retrieved pain reduction did not differ between groups. We did not aim to investigate differences in neonatal outcome between groups or between women receiving meptazinol with the entire cohort. Thus, the here-detected differences can only be presented descriptively.

## Conclusion

Our data showed an equieffective pain reduction by both intramuscular (Meptazinol^IM^) and intravenous (Meptazinol^IV^) administration of meptazinol with similar rates of side effects. Although we did see a higher usage of dimenhydrinate for treating emesis in the Meptazinol^IV^ group, we conclude that both routes of administration of meptazinol are equivalent and patients with a history of hyperemesis or motion sickness should be considered for intramuscular administration. The comparably poorer perinatal outcome hinders us to confirm that meptazinol is safe and can be recommended without restrictions. Further studies are needed to compare perinatal outcome data in regard to drugs applied for pain relief.

### Supplementary Information

Below is the link to the electronic supplementary material.

Supplementary file1 (PDF 598 KB)

Supplementary file2 (DOCX 22 KB)

## Data Availability

The datasets used and/or analyzed during the current study are available from the corresponding author on reasonable request.

## Abbreviation

NRS: Numeric rating scale
