# Supplementary material for: SPAM—sub partual analgesia with meptazinol: a prospective cohort study comparing intramuscular with intravenous administration
Source: Arch Gynecol Obstet. 2023 May 9;309(5):1873–81. doi: 10.1007/s00404-023-07056-y (PMC11018690; doi:10.1007/s00404-023-07056-y)
Supplement: Supplementary file 1 — Supplementary file1 (PDF 598 KB) [file 404_2023_7056_MOESM1_ESM.pdf]

SPAM-CRF: Meptid®-Application Observation

Preconditions (*please tick!*)

- ☐ Singleton pregnancy
- ☐ ≥ 37+0 weeks of gestation
- ☐ Patient in 1st stage of labor according to cervical findings
- ☐ No prior administration of Meptid
- ☐ No contraindications for Meptazinol (*asthma, emphysema, heart diesease, myxedema*)
- ☐ No contraindications against intramuscular injections (*bleeding tendency, therap. anticoagulation*)
- ☐ CTG control over 30 minutes FIGO normal
- ☐ Runngin CTG (at least 30 min) assigned in Huntleigh
- ☐ No chronic pain medication use before/in pregnancy

Bishop Score <u>BEFORE</u> administration (*<u>Administration only in 1st stage of labor)</u>*)

Findings	0 points	1 point	2 points	3 points	Total
Cervical effacement	0–30 %	40–50 %	60–70 %	> 80 %	
Cervical consistency	Solid	Medium	Soft	-	
Cervix position	Sacral	Mediosacral	Centered	-	
Cervical dilation	Closed	1–2 cm	3–4 cm	> 5 cm	
Height of the preceding part relative to the interspinal plane	- 3	- 2	- 1	>= 0	
				Summe Bishop Score:	

Pain score <u>BEFORE</u> administration (*please tick!*)

Ask the patient for the number on the scale from 0 to 10 that the patient feels is the maximum pain of the contraction, BEFORE you administer the pain medication (Meptazinol)

0	1	2	3	4	5	6	7	8	9	10

„0“ means freedom from pain — *„10“ means strongest pain imaginable*

Application way:

- ☐ intramuscular (*weight adapted dosage*)
- ☐ intravascular (weight adapted dosage in 250ml NaCl i.v. by Infusomat over a period of 30 min)

Cave: <u>No administration of other drugs in parallel with meptazinol</u> (over 1 h after meptazinol administration)

Current weight pregnant women:	|______________| kg
Dosage Meptazinol:	|______________| mg
Administration time Meptazinol:	|______________| o'clock

Body weight (kg) pregnant woman	Meptazinol in mg	Meptazinol in ml if 1 ml = 100 mg (e.g. Meptid®)
50	100	1
60	120	1,2
70	140	1,4
80	160	1,6
90	180	1,8
100	200	2,0
110	220	2,2
120	240	2,4

☐ *CTG written continuously for 60 minutes after administration and assigned in Huntleigh*

Pain Score <u>1 hour AFTER</u> administration (*please tick!*)

Ask the patient for the number on the scale from 0 to 10 that the patient feels is the maximum pain of the contraction, AFTER you administered the pain medication (Meptazinol)

0	1	2	3	4	5	6	7	8	9	10

„0“ means freedom from pain — *„10“ means strongest pain imaginable*

UNIVERSITÄTS KLINIKUM jena

SPAM: Meptid®-Application Observation

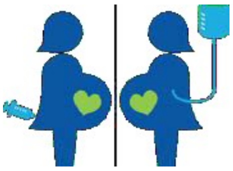

Side effects (*please tick!*)

☐ Nausea
☐ Emesis
☐ Fatigue
☐ Vertigo
☐ Perspiration
☐ Headache
☐ Gastrointestinal disturbance (abdominal pain, obstipation, diarrhea)
☐ Hallucinatins (Perception of non-existent things /sounds)
☐ Hypersensitivity reactions (rashes, itching)
☐ Change in blood pressure (increase or decrease)
☐ Other: |____|

Other pain medications during childbirth

Non-opioid ☐ No non-opioids used

	application				time				
	p.o.	i.v.	i.m.	supp.	Dilation	Expulsion	Afterbirth	date	time
Butylscopolamin (Buscopan®)	☐	☐	☐	☐	☐	☐	☐		
Paracetamol	☐	☐	☐	☐	☐	☐	☐		

Inhalation analgesia ☐ No inhalation analgesia used

	time				
	Dilation	Expulsion	Afterbirth	date	time
Dinitrogen monoxide (Laughing gas, Livopan®)	☐	☐	☐		

Opioid ☐ No opioids used

	Application		time				
	i.v.	i.m.	Dilation	Expulsion	Afterbirth	date	time
Fentanyl	☐	☐	☐	☐	☐		

Regional Analgesia ☐ No regional analgesia used

		Time	
		date	time
Peridural anaesthesia (PDA)	☐		
Peripheral block (e.g. pudendal block)	☐		

Outcome

Nr. In birth book: |________________|

Birth details	Child Outcome
☐ Induction<br>☐ Spontanous<br>☐ Vaginal Operative Delivery<br>☐ C-Section<br>☐ Oxytocin sub partu: increased to |____________| IE	Date of birth: |______________________| Time of birth: |______________________| Birth weight: |______________________| ☐ Respiratory distress syndrom<br>☐ disorder CPAP

Satisfaction with pain treatment sub partu (*please tick BEFORE transfer to the maternity ward!*)

After the birth, ask the patient the number on the scale from 0 to 10 that reflects her satisfaction with the pain management.

0	1	2	3	4	5	6	7	8	9	10

„0"means completely dissatisfied *„10"means very satisfied*

☐ *QUIPS consent form handed over to patient*

Put the completed form in the birth book!
