# Supplementary material for: SPAM—sub partual analgesia with meptazinol: a prospective cohort study comparing intramuscular with intravenous administration
Source: Arch Gynecol Obstet. 2023 May 9;309(5):1873–81. doi: 10.1007/s00404-023-07056-y (PMC11018690; doi:10.1007/s00404-023-07056-y)
Supplement: Supplementary file 2 — Supplementary file2 (DOCX 22 KB) [file 404_2023_7056_MOESM2_ESM.docx]

| Supplement S2 – QUIPS Data according to the original QUIPS questionnaire | | | | |
| --- | --- | --- | --- | --- |
|  | Entire cohort (n=78) | IM Group (n=38) | IV Group (n=40) | p value |
| Previous Obstetric History | 21.8% | 21.1% | 22.5% | 0.877 |
| Prior C-Section | 9% | 10.5% | 7.5% | 0.708 |
| Opioid premedication | 54.5% | 57.9% | 41.1% | 0.456 |
| Accompanying person present | 91.1% | 89.5% | 94.4% | 0.542 |
| Childbirth preparation |  |  |  |  |
| None | 12.8% | 13.2% | 12.5% | 0.931 |
| Antenatal class | 56.4% | 50% | 62.5% | 0.266 |
| Acupuncture | 19.2% | 23.7% | 15% | 0.331 |
| Perineal massage | 33.3% | 42.1% | 25% | 0.109 |
| Belly massage | 23.1% | 13.2% | 32.5% | **0.043** |
| Herbal teas | 53.8% | 60.5% | 47.5% | 0.249 |
| Meditation | 5.1% | 5.3% | 5% | 0.958 |
| Sitting baths | 21.8% | 21.1% | 22.5% | 0.877 |
| Homeopathy | 3.8% | 0% | 7.5% | 0.085 |
| Pregnancy gymnastics | 26.9% | 31.6% | 22.5% | 0.366 |
| Hypnosis | 3.8% | 5.3% | 2.5% | 0.526 |
| Flaxseed | 17.9% | 18.4% | 17.9% | 0.916 |
| Involvement in decision making | 92.3% | 94.7% | 90% | 0.433 |
| Personal view on pain medication utilization before birth |  |  |  |  |
| No pain medication | 17.9% | 13.2% | 22.5% | 0.283 |
| Independent decision during birth | 64.1% | 68.4% | 60% | 0.438 |
| Doctor’s/Midwife’s decision | 9% | 10.5% | 7.5% | 0.640 |
| Definitely pain medication | 17.9% | 18.4% | 17.5% | 0.916 |
| View on pain medication changed after delivery | 21.8% | 23.7% | 20% | 0.694 |
| Complications during 3rd stage of labor | 9% | 10.5% | 7.5% | 0.640 |
| Duration of maximum pain* | 2 (1/2.75) | 2 (1/2.75) | 2 (1/2.75) | 0.725 |
| Time of worst pain |  |  |  |  |
| Start of pain | 9% | 10.5% | 7.5% | 0.640 |
| During examination | 5.1% | 5.3% | 5% | 0.958 |
| During contractions | 70.5% | 60.5% | 80% | 0.059 |
| During pushing | 43.6% | 52.6% | 35% | 0.117 |
| As child was being born | 25.6% | 31.6% | 20% | 0.242 |
| During 3^rd^ stage of labor | 1.3% | 2.6% | 0% | 0.302 |
| During suturing of birth injuries | 1.3% | 2.6% | 0% | 0.302 |
| After Delivery | 1.3% | 0% | 2.5% | 0.327 |
| Non pharmaceutical pain relief used | 65.4% | 68.4% | 62.5% | 0.583 |

Data are n (%) or median and interquartile range (IQR) unless otherwise specified. Significant findings (p<0.05) are highlighted in bold. Cohort differs due to missing cases (n=54), Meptazinol^IM^ – subgroup with intramuscular application of meptazinol; Meptazinol^IV^ subgroup with intravenous application of meptazinol; Duration of maximum pain* - 0: none, 1: bearably long, 2: too long, 3: unbearably long
